# Supplementary material for: Parametric and nonparametric propensity score weighting analysis with subgroup covariate balance
Source: Stat Methods Med Res. 2026 Jan 29;35(4):736–51. doi: 10.1177/09622802251415157 (PMC13161500; doi:10.1177/09622802251415157)
Supplement: sj-pdf-1-smm-10.1177_09622802251415157 - Supplemental material for Parametric and nonparametric propensity score weighting analysis with subgroup covariate balance [file sj-pdf-1-smm-10.1177_09622802251415157.pdf]

Online Supplementary Materials for
"Parametric and Nonparametric Propensity Score Weighting Analysis with Subgroup Covariate Balance"

1 Subgroup Tuning Algorithm

Algorithm 1 : Choosing σ in kG-SBPS

1: For each $\sigma \in \{\sigma_1, \ldots, \sigma_{20}\}$.

2: Compute IPW: $\{\hat{p}_{\boldsymbol{\theta}}(\boldsymbol{\omega}_{l_{99}}(\boldsymbol{Z})_i, \boldsymbol{S}_i); i = 1, 2, ..., N\}$ using the kG-SBPS in Section 2.3 and 2.4.

3: Compute $w_{i1} = 1/\hat{p}_{\boldsymbol{\theta}}(\boldsymbol{\omega}_{l_{99}}(\boldsymbol{Z})_i, \boldsymbol{S}_i)$ and $w_{i0} = 1/(1 - \hat{p}_{\boldsymbol{\theta}}(\boldsymbol{\omega}_{l_{99}}(\boldsymbol{Z})_i, \boldsymbol{S}_i))$ for ATE estimation or $w_{i1} = 1$ and $w_{i0} = \hat{p}_{\boldsymbol{\theta}}(\boldsymbol{\omega}_{l_{99}}(\boldsymbol{Z})_i, \boldsymbol{S}_i)/(1 - \hat{p}_{\boldsymbol{\theta}}(\boldsymbol{\omega}_{l_{99}}(\boldsymbol{Z})_i, \boldsymbol{S}_i))$ for ATT estimation.

4: Evaluate global balance: calculate the S/D of each observed covariates $\boldsymbol{Z}$ in the overall population such that

$$\boldsymbol{B}_{\text{global}} = 100 \times \left| \frac{\sum_{i=1}^{N} w_{i1} \boldsymbol{Z}_i T_i}{\sum_{i=1}^{N} w_{i1} T_i} - \frac{\sum_{i=1}^{N} w_{i0} \boldsymbol{Z}_i (1 - T_i)}{\sum_{i=1}^{N} w_{i0} (1 - T_i)} \right| / sd(\boldsymbol{Z}),$$

where $sd(\boldsymbol{Z})$ are the pooled standard deviations of $\boldsymbol{Z}$ between two treatment groups such that $sd(\boldsymbol{Z}) = \sqrt{(sd_{(1)}^2 + sd_{(0)}^2)/2}$, where $sd_{(1)}$ and $sd_{(0)}$ are the weighted standard deviations of $\boldsymbol{Z}$

$$sd_{(1)}^2 = \frac{\sum w_{i1} T_i}{(\sum w_{i1} T_i)^2 - \sum w_{i1}^2 T_i} \sum w_{i1} T_i \left(\boldsymbol{Z}_i - \frac{w_{i1} \boldsymbol{Z}_i T_i}{\sum w_{i1} Ti} \right)^2$$

$$sd_{(0)}^2 = \frac{\sum w_{i0} (1 - T_i)}{(\sum w_{i0} (1 - T_i))^2 - \sum w_{i0}^2 (1 - T_i)} \sum w_{i0} (1 - T_i) \left(\boldsymbol{Z}_i - \frac{w_{i0} \boldsymbol{Z}_i (1 - T_i)}{\sum w_{i0} (1 - Ti)} \right)^2.$$

5: Evaluate subgroup balance: calculate the S/D of the observed covariates in the covariate-defined subpopulations such that

$$\boldsymbol{B}_{S_k} = 100 \times \left| \frac{\sum w_{i1} \boldsymbol{Z}_i T_i}{\sum w_{i1} T_i} - \frac{\sum w_{i0} \boldsymbol{Z}_i (1 - T_i)}{\sum w_{i0} (1 - T_i)} \right| / sd_k(\boldsymbol{Z}) \,\Big|_{S_{ik}=1},$$

where $S_k = \{i | S_{ik} = 1\}$. And $sd_k(\boldsymbol{Z})$ is calculated from subjects in each subpopulation.

6: Output $l^* = \arg\min \frac{1}{MK} \sum_{m=1}^{M} \sum_{k=1}^{K} \boldsymbol{B}_{S_k}$ subject to $\max(\boldsymbol{B}_{\text{global}}) \leq 10\%$. Output "fail" if $\max(\boldsymbol{B}_{\text{global}}) > 10\%$.

2 Proof of consistency of the proposed ATE and ATT estimators

2.1 ATE estimator

We aim to show that the estimator $\hat{\boldsymbol{\theta}}$ obtained by maximizing the loss function $L_{ATE}(\boldsymbol{\theta})$ (Equation 2 in the main paper) is consistent for the true parameter $\boldsymbol{\theta}_0$, under the assumption that the propensity score model $p_{\boldsymbol{\theta}}$ is correctly specified.

The estimator $\hat{\boldsymbol{\theta}}$ maximizes the sample objective

$$Q_N(\boldsymbol{\theta}) = \frac{1}{N} L_{ATE}(\boldsymbol{\theta}), \tag{1}$$

where

$$\begin{aligned} L_{ATE} = &\sum_{i=1}^{N} T_i \left[\log\left(\frac{p_{\boldsymbol{\theta}}(\boldsymbol{X}_i)}{1-p_{\boldsymbol{\theta}}(\boldsymbol{X}_i)} \right) - \frac{1}{p_{\boldsymbol{\theta}}(\boldsymbol{X}_i)} \right] \\ &+ \sum_{i=1}^{N} (1-T_i) \left[\log\left(\frac{1-p_{\boldsymbol{\theta}}(\boldsymbol{X}_i)}{p_{\boldsymbol{\theta}}(\boldsymbol{X}_i)} \right) - \frac{1}{1-p_{\boldsymbol{\theta}}(\boldsymbol{X}_i)} \right]. \end{aligned} \tag{2}$$

The corresponding population objective function is:

$$\begin{aligned} Q(\boldsymbol{\theta}) = \mathbb{E}\Bigg[&T \left(\log \frac{p_{\boldsymbol{\theta}}(\boldsymbol{X})}{1-p_{\boldsymbol{\theta}}(\boldsymbol{X})} - \frac{1}{p_{\boldsymbol{\theta}}(\boldsymbol{X})} \right) \\ &+ (1-T) \left(\log \frac{1-p_{\boldsymbol{\theta}}(\boldsymbol{X})}{p_{\boldsymbol{\theta}}(\boldsymbol{X})} - \frac{1}{1-p_{\boldsymbol{\theta}}(\boldsymbol{X})} \right) \Bigg]. \end{aligned} \tag{3}$$

We assume the propensity score model is correctly specified, i.e., there exists a true parameter $\boldsymbol{\theta}_0$ such that $p_{\boldsymbol{\theta}_0}(\boldsymbol{X}) = \mathbb{P}(T = 1 \mid \boldsymbol{X})$.

We prove that $\hat{\boldsymbol{\theta}} \xrightarrow{p} \boldsymbol{\theta}_0$ by verifying the following three conditions:

(i) Uniform Convergence. By Law of Large Numbers, $Q_N(\boldsymbol{\theta})$ converges uniformly in probability to $Q(\boldsymbol{\theta})$:

$$\sup_{\boldsymbol{\theta} \in \Theta} |Q_N(\boldsymbol{\theta}) - Q(\boldsymbol{\theta})| \xrightarrow{p} 0.$$

This holds under standard regularity conditions:

- The parameter space Θ is compact;
- The function $p_{\boldsymbol{\theta}}(\boldsymbol{X})$ is continuous in $\boldsymbol{\theta}$ and bounded away from 0 and 1;
- The summands in $Q_N(\boldsymbol{\theta})$ are measurable and dominated by an integrable envelope function.

(ii) Identification. We show that the population objective function $Q(\boldsymbol{\theta})$ is uniquely maximized at $\boldsymbol{\theta}_0$. Writing $p = p_{\boldsymbol{\theta}}(\boldsymbol{X})$ and $p_0 = p_{\boldsymbol{\theta}_0}(\boldsymbol{X})$, the integrand of $Q(\boldsymbol{\theta})$ becomes:

$$\ell(p) = p_0 \left(\log \frac{p}{1-p} - \frac{1}{p} \right) + (1-p_0) \left(\log \frac{1-p}{p} - \frac{1}{1-p} \right). \tag{4}$$

Taking first derivative with respect to p:

$$\frac{\partial \ell(p)}{\partial p} = \frac{p_0 - p}{p^2(1-p)^2} = 0 \tag{5}$$

It is straightforward to verify that $\ell(p)$ is uniquely maximized at $p = p_0$, and thus $Q(\boldsymbol{\theta})$ is uniquely maximized at $\boldsymbol{\theta} = \boldsymbol{\theta}_0$ due to the correct specification of the model.

(iii) Existence of $\hat{\boldsymbol{\theta}}$. Under continuity of $Q_N(\boldsymbol{\theta})$ and compactness of Θ, the maximizer $\hat{\boldsymbol{\theta}}$ exists.

Hence, the proposed ATE estimator for the propensity score is consistent under correct propensity model specification. Furthermore, the discussion above shows that this estimator is an M-estimator, which possesses the typical parametric convergence rate with $\sqrt{N}$-consistency.

2.2 ATT estimator

We now consider the consistency of the ATT estimator, which is obtained by maximizing the following loss function:

$$L_{ATT}(\boldsymbol{\theta}) = \sum_{i=1}^{N} \left\{ T_i \log\left(\frac{p_{\boldsymbol{\theta}}(\boldsymbol{X}_i)}{1 - p_{\boldsymbol{\theta}}(\boldsymbol{X}_i)}\right) - \frac{1 - T_i}{1 - p_{\boldsymbol{\theta}}(\boldsymbol{X}_i)} \right\}, \quad (6)$$

where $p_{\boldsymbol{\theta}}(\boldsymbol{X})$ is modeled by logit link and assume the propensity score is correctly specified.

Following the same logic presented in the proof of ATE estimator, we just need to verify the "Identification" condition using $L_{ATT}(\boldsymbol{\theta})$. It straightforward to verify that the integrand of the expectation of $L_{ATT}(\boldsymbol{\theta})$ is uniquely maximized at $p = p_0$, because:

- The term $T \log\left(\frac{p}{1-p}\right)$ corresponds to a log-likelihood contribution.
- The term $-\frac{1-T}{1-p}$ penalizes deviations when $T = 0$.

3 Supplemental Tables and Figures

Table S1: The performance of various methods to estimate **subgroup ATTs** in the simulations with the **correct PS model (PS1, see Section 3.1)**. The true treatment effect for subgroup 1 to 4 are -10, $-10/3$, $10/3$, and 10, respectively. The number of Monte Carlo repetitions is 500.

	**Outcome**	**Subgroup**	**Logistic**	**Logistic-S**	**CBPS**	**SBPS**	**G-SBPS**	**kG-SBPS**
%Bias	Standard	**1**	-0.23	-0.12	-0.59	-0.12	-0.10	0.22
		**2**	-3.88	0.57	-4.93	0.57	-0.18	-0.25
		**3**	-1.29	0.15	0.01	0.15	0.23	0.59
		**4**	-0.99	-0.54	-0.70	-0.54	-0.02	0.67
	Extended	**1**	-1.61	-0.95	-1.28	-0.95	-1.09	-0.62
		**2**	-3.38	-0.57	-3.42	-0.57	-0.75	-0.35
		**3**	0.24	2.46	1.23	2.46	2.40	-0.07
		**4**	-1.37	-0.98	-1.18	-0.98	-0.39	0.15
RMSE	Standard	**1**	2.78	0.34	3.08	0.34	0.14	0.24
		**2**	1.99	0.42	2.22	0.42	0.11	0.17
		**3**	2.01	0.58	2.09	0.58	0.10	0.16
		**4**	2.15	0.84	1.67	0.84	0.10	0.25
	Extended	**1**	2.82	1.11	3.02	1.11	1.12	0.34
		**2**	1.84	0.98	2.02	0.98	0.94	0.25
		**3**	1.98	1.01	2.02	1.01	0.82	0.25
		**4**	2.70	1.44	2.18	1.44	0.91	0.42

Table S2: The performance of various methods to estimate **subgroup ATTs** in the simulations with the **misspecified PS model (PS2, see Section 3.1)**. The true treatment effect for subgroup 1 to 4 are -10, $-10/3$, $10/3$, and 10, respectively. The number of Monte Carlo repetitions is 500.

	**Outcome**	**Subgroup**	**Logistic**	**Logistic-S**	**CBPS**	**SBPS**	**G-SBPS**	**kG-SBPS**
%Bias	Standard	**1**	-241.55	-34.98	-299.36	-34.98	-3.40	-13.31
		**2**	-285.46	-34.71	-464.46	-34.71	0.01	-7.35
		**3**	-267.60	-80.45	-112.08	-80.17	-0.15	-11.35
		**4**	-165.89	-55.06	-123.82	-55.06	-0.10	-11.36
	Extended	**1**	45.82	29.88	39.41	29.88	21.15	7.62
		**2**	2.08	96.21	-79.89	96.21	105.05	6.36
		**3**	-293.19	-148.20	-173.98	-148.02	-81.50	-15.65
		**4**	-206.03	-84.18	-162.27	-84.18	-18.31	-17.92
RMSE	Standard	**1**	24.27	3.81	30.06	3.81	0.50	1.79
		**2**	10.03	1.38	15.86	1.38	0.12	0.57
		**3**	9.16	2.80	4.36	2.80	0.10	0.60
		**4**	16.68	5.79	12.47	5.79	0.16	1.45
	Extended	**1**	5.41	3.85	4.97	3.85	2.44	1.12
		**2**	2.45	3.50	3.75	3.50	3.74	0.46
		**3**	9.97	5.10	6.18	5.10	2.90	0.71
		**4**	20.71	8.83	16.33	8.83	2.10	2.18

Table S3: The performance of various methods to estimate **subgroup ATTs** in the simulations with the **misspecified PS model (PS2, see Section 3.1)**. The simulation setting is the same as Table S11 except that the sample size per subgroup is doubled at $N_k = 1000$.

	**Outcome**	**Subgroup**	**Logistic**	**Logistic-S**	**CBPS**	**SBPS**	**G-SBPS**	**kG-SBPS**
%Bias	Standard	**1**	-241.72	-33.73	-299.05	-33.73	-11.76	-8.71
		**2**	-275.92	-33.55	-450.34	-33.55	-1.76	-5.63
		**3**	-257.87	-78.71	-106.73	-78.48	-0.31	-6.72
		**4**	-165.83	-53.84	-124.16	-53.84	0.08	-7.68
	Extended	**1**	46.60	29.03	40.87	29.03	22.57	5.57
		**2**	11.55	99.93	-67.43	99.93	108.39	6.97
		**3**	-283.78	-145.66	-168.21	-145.53	-80.29	-13.09
		**4**	-205.95	-83.08	-162.64	-83.08	-17.65	-13.55
RMSE	Standard	**1**	24.23	3.54	29.97	3.54	1.32	1.05
		**2**	9.46	1.23	15.21	1.23	0.13	0.36
		**3**	8.74	2.68	3.94	2.68	0.08	0.35
		**4**	16.63	5.54	12.46	5.54	0.11	0.94
	Extended	**1**	5.07	3.37	4.59	3.37	2.44	0.74
		**2**	1.87	3.49	2.97	3.49	3.75	0.38
		**3**	9.59	4.94	5.85	4.94	2.76	0.53
		**4**	20.65	8.55	16.32	8.55	1.94	1.56

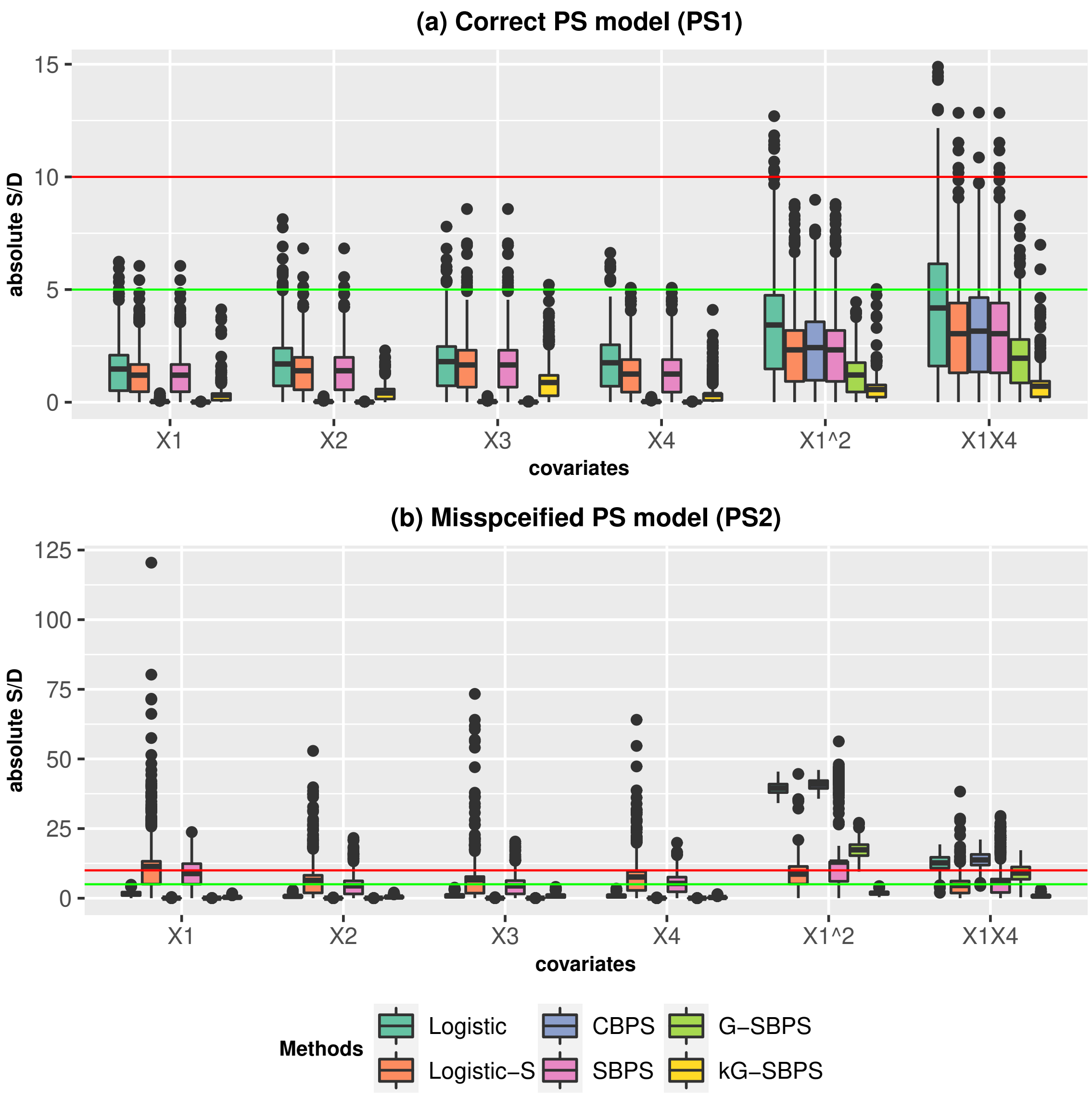

Figure S1. Boxplots of the standardized differences (S/D; %) in the overall population when estimating the **ATE** in the simulation studies. The data are simulated from the correct PS model (PS1) or the misspecified PS model (PS2). The boxplots show the distribution of S/D from 500 Monte Carlo repetitions. The red and green horizontal lines mark the 10% and 5% S/D, respectively.

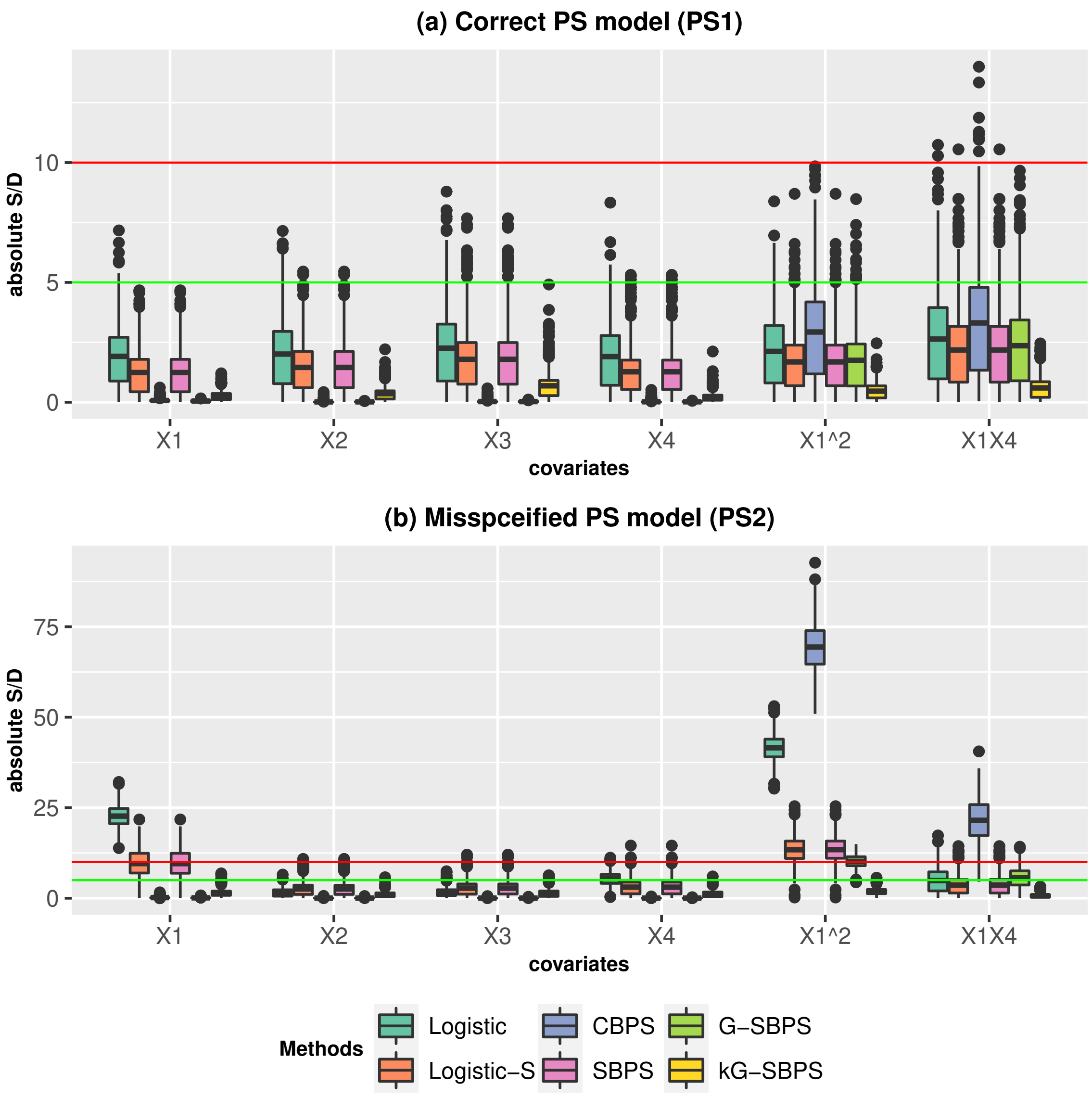

Figure S2. Boxplots of the standardized differences (S/D; %) in the overall population when estimating the **ATT** in the simulation studies. The data are simulated from the correct PS model (PS1) or the misspecified PS model (PS2). The boxplots show the distribution of S/D from 500 Monte Carlo repetitions. The red and green horizontal lines mark the 10% and 5% S/D, respectively.

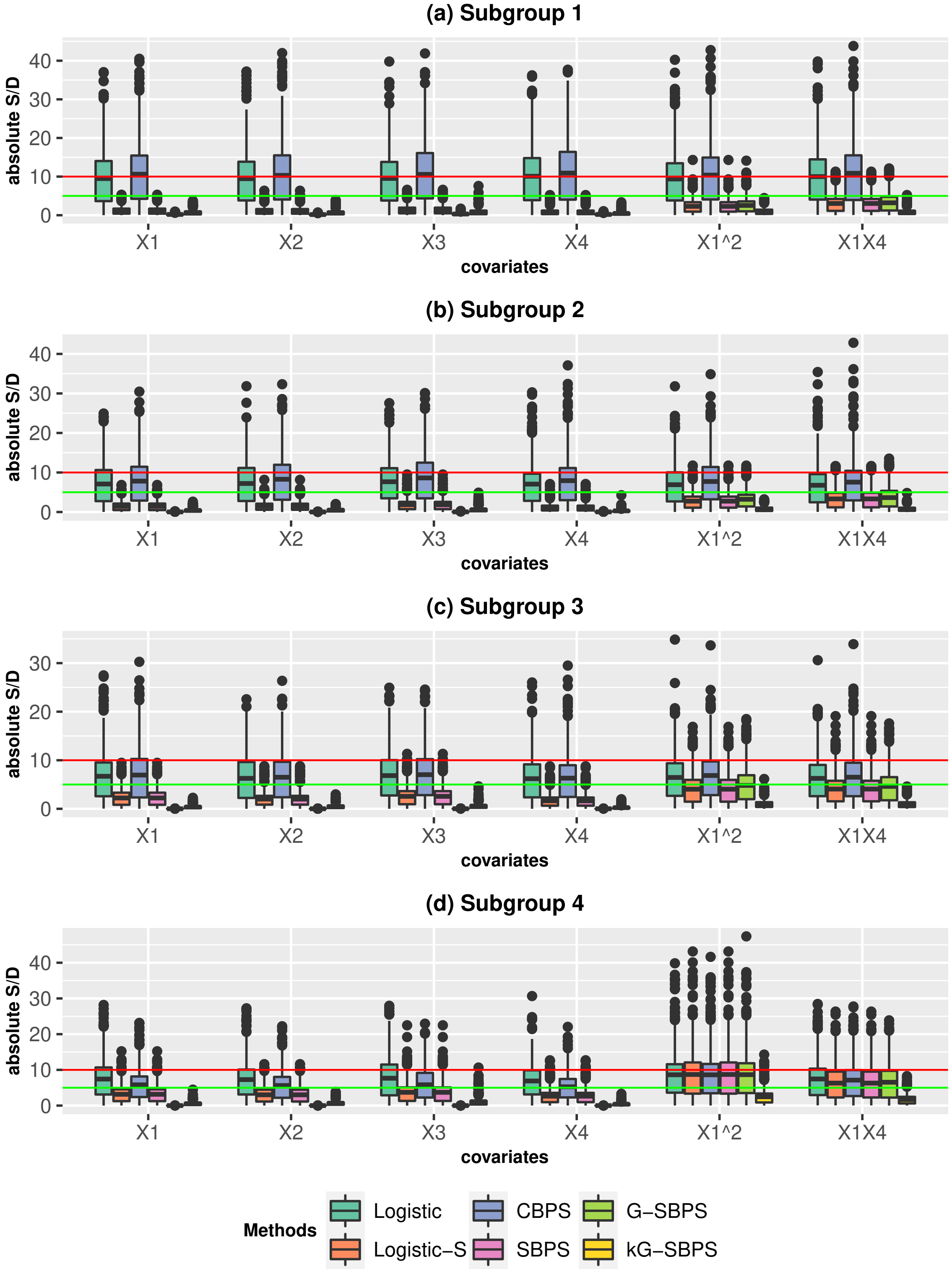

Figure S3. Boxplots of the subgroup S/D in the estimation of **ATT** from the simulations. The data are simulated from the correct PS model (PS1; see Section 3.1). The boxplots show the distribution of S/D from 500 Monte Carlo repetitions. The red and green horizontal lines mark the 10% and 5% S/D, respectively.

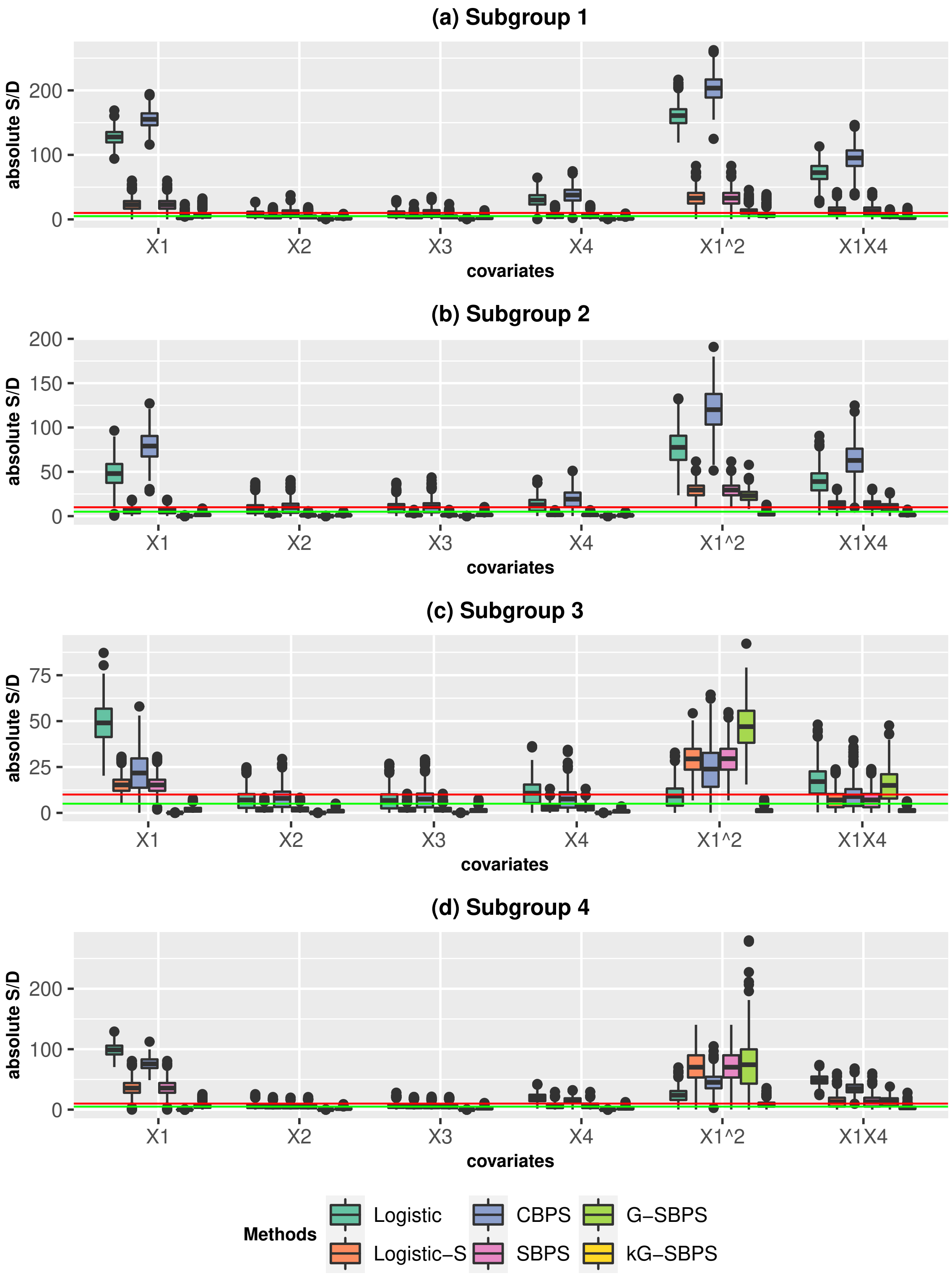

Figure S4. Boxplots of the subgroup S/D in the estimation of **ATT** from the simulations. The data are simulated from the misspecified PS model (PS2; see Section 3.1). The boxplots show the distribution of S/D from 500 Monte Carlo repetitions. The red and green horizontal lines mark the 10% and 5% S/D, respectively.

Table S4: Description of the subgroups in the RHC data application. N_0: the number of non-RHC patients; N_1 the number of RHC patients.

Subgroup	Description	Sample Size N_0	N_1
3 subgroups (Non-overlapped)	Mean Blood Pressure		
	subgroup 1: < 80	2028	1690
	subgroup 2: $80 \geq$ and ≤ 120	679	229
	subgroup 3: > 120	844	264
6 subgroups (Overlapped)	Mean Blood Pressure		
	subgroup 1: < 80	2028	1690
	subgroup 2: $80 \geq$ and ≤ 120	679	229
	subgroup 3: > 120	844	264
	Estimated Probability of Surviving 2 Months		
	subgroup 4: < 0.3	302	238
	subgroup 5: ≥ 0.3 and ≤ 0.7	1910	1296
	subgroup 6: > 0.7	1339	649

Table S5: The estimated subgroup ATEs in the RHC data using the different propensity score analysis methods. The treatment effect measures the average increase in the hospital length of stay in days between RHC and non-RHC.

**Subgroup**		**Logistic**	**Logistic-S**	**CBPS**	**SBPS**	**G-SBPS**	**kG-SBPS**
3 subgroups (Non-overlapped)	subgroup 1	1.77	1.98	1.26	1.98	1.69	1.75
	subgroup 2	5.65	1.97	5.95	1.97	1.61	1.13
	subgroup 3	4.88	3.47	4.89	3.47	2.34	3.38
6 subgroups (Overlapped)	subgroup 1	1.71	-	1.21	-	1.28	1.05
	subgroup 2	5.46	-	5.58	-	0.84	1.48
	subgroup 3	4.92	-	4.84	-	2.70	3.10
	subgroup 4	4.35	-	3.64	-	3.86	2.32
	subgroup 5	2.51	-	2.05	-	0.58	1.82
	subgroup 6	3.03	-	3.12	-	2.34	0.87

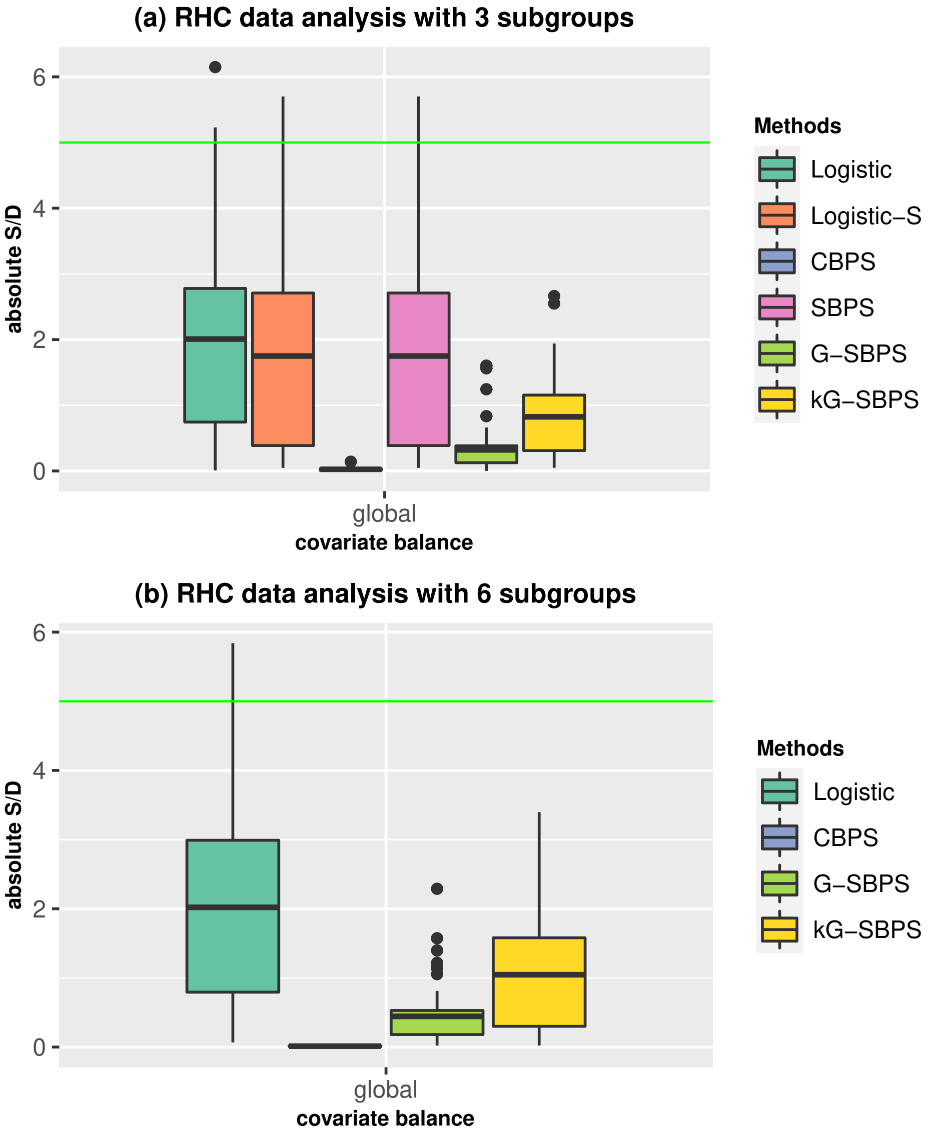

Figure S5. Boxplots of the standardized differences (S/D) of all covariates in the RHC data analysis. Green line: 5% S/D. The S/D is calculated for the overall population ("global" covariance balance).

Table S6: Baseline covariates of subjects in the DSMT data. Mean (standard deviation) is reported for age and percent of residents living below poverty. Count (percentage) is reported for all other categorical variables.

**Covariate**	**Untreated** $n = 16401$	**DSMT** $n = 3283$
**Age (in years)**	75.3 (7.2)	75.3 (6.3)
**Percent of residents living below poverty (%)**	14.6 (9.3)	15.2 (9.5)
**Female sex** - n(%)	8048 (49.1)	1611 (49.1)
**Type II diabetes** - n(%)	15891 (96.9)	3181 (96.9)
**Type I diabetes** - n(%)	516 (3.1)	106 (3.2)
**Incident diabetes** - n(%)	2450 (14.9)	490 (14.9)
**Race** (Hispanic) - n(%)		
White	11410 (69.6)	2168 (66.0)
Black	1931 (11.8)	375 (11.4)
**Cancer category** (Colorectal cancer) - n(%)		
Prostate cancer	6763 (41.2)	1393 (42.4)
Breast cancer	6717 (41.0)	1340 (40.8)
**Married** - n(%)	6855 (41.8)	1503 (45.8)
**Medicare original entitlement status** - n(%)	1301 (7.9)	277 (8.4)
**Elixhauser comorbidity index category** $(0-1)$ - n(%)		
$1-2$	5922 (36.1)	1254 (38.2)
> 3	6723 (41.0)	1250 (38.1)
**Quantile of zip code percent non-high school graduates** (Very poor) - n(%)		
Very good	4202 (25.6)	811 (24.7)
Good	3924 (23.9)	707 (21.5)
Poor	3602 (22.0)	731 (22.3)
**Medicaid dual eligible during index year** - n(%)	2380 (14.5)	589 (17.9)
**Primary care provider in the year prior the index year** - n(%)	9202 (56.1)	1601 (48.8)
**Living in metropolitan** - n(%)	2959 (18.0)	692 (21.1)
**Diabetes complication category** (No complication)- n(%)		
Renal manifestation	1677 (10.2)	386 (11.8)
Ophthalmic manifestation	2069 (12.6)	567 (17.3)
Neurological manifestation	3618 (22.1)	941 (28.7)
Unspecified	1094 (6.7)	267 (8.1)
**Elixhauser comorbidity category** - n(%)		
Alcohol abuse	195 (1.2)	46 (1.4)
Arrhythmia	4289 (26.2)	847 (25.8)
Blood loss Anemia	379 (2.3)	70 (2.1)
Congestive heart failure	2964 (18.1)	543 (16.5)
COPD (chronic obstructive pulmonary disease)	4092 (24.9)	688 (21.0)
Coagulopathy	802 (4.9)	158 (4.8)
Deficiency anemia	2570 (15.7)	444 (13.5)
Depression	2639 (16.1)	432 (13.2)
Drug abuse	224 (1.4)	34 (1.0)
Fluid and electrolyte disorders	2801 (17.1)	496 (15.1)
AIDS/HIV	12 (0.1)	NA*
Hypothyroidism	4612 (28.2)	864 (26.3)
Liver disease	1224 (7.5)	259 (7.9)
Obesity	2531 (15.4)	557 (17.0)
Other neurological disorders	1403 (8.6)	187 (5.7)
Pulmonary circulation disorders	674 (4.1)	120 (3.7)
Peptic ulcer disease excluding bleeding	252 (1.5)	50 (1.5)
Peripheral vascular disorders	3580 (21.8)	626 (19.1)
Paralysis	275 (1.7)	41 (1.2)
Psychoses	475 (2.9)	61 (1.9)
Renal failure	3010 (18.4)	680 (20.7)
Rheumatoid arthritis	1057 (6.4)	187 (5.7)
Valvular disease	2513 (15.3)	500 (15.2)
Weight loss	875 (5.3)	122 (3.7)
Hypertension	14152 (86.3)	2892 (88.1)

*The AIDS/HIV in DSMT group contains fewer than 11 patients. It was not reported because of the CMS cell size suppression policy to protect the confidentiality of entrollees.

Table S7: Description of the subgroups in the DSMT data application. N_0: the number of DSMT patients; N_1 the number of non-DSMT patients.

**Subgroup**	**Description**	**Sample Size** N_0	N_1
4 subgroups (Non-overlapped)	Living area and Medicaid dual eligibility during index year		
	subgroup 1: Non-metropolitan and Non-eligible	11599	2201
	subgroup 2: Non-metropolitan and Eligible	1843	390
	subgroup 3: Metropolitan and Non-eligible	2422	493
	subgroup 4: Metropolitan and Eligible	537	199
6 subgroups (Overlapped)	Living area and Medicaid dual eligibility during index year		
	subgroup 1: Non-metropolitan and Non-eligible	11599	2201
	subgroup 2: Non-metropolitan and Eligible	1843	390
	subgroup 3: Metropolitan and Non-eligible	2422	493
	subgroup 4: Metropolitan and Eligible	537	199
	Marriage status		
	subgroup 5: Not married	9546	1780
	subgroup 6: Married	6855	1503

Table S8: The estimated subgroup ATEs in the DSMT data using the different propensity score analysis methods. The treatment effect measures the average increase in the hospitalization rate (%) within 3 years of index year between DSMT and non-DSMT.

**Subgroup**		**Logistic**	**Logistic-S**	**CBPS**	**SBPS**	**G-SBPS**	**kG-SBPS**
4 subgroups (Non-overlapped)	subgroup 1	-3.8	-4.0	-3.8	-4.0	-4.0	-3.8
	subgroup 2	-12.6	-6.4	-12.6	-6.4	-6.6	-5.4
	subgroup 3	-4.3	-8.8	-4.3	-8.8	-9.1	-9.4
	subgroup 4	-26.2	-15.1	-26.5	-15.1	-16.9	-19.1
6 subgroups (Overlapped)	subgroup 1	-3.8	-	-3.7	-	-3.9	-3.9
	subgroup 2	-12.6	-	-12.7	-	-6.6	-5.5
	subgroup 3	-4.3	-	-4.0	-	-9.7	-9.1
	subgroup 4	-26.2	-	-27.2	-	-17.6	-19.7
	subgroup 5	-10.4	-	-10.5	-	-8.3	-8.5
	subgroup 6	-1.4	-	-1.2	-	-3.1	-2.7

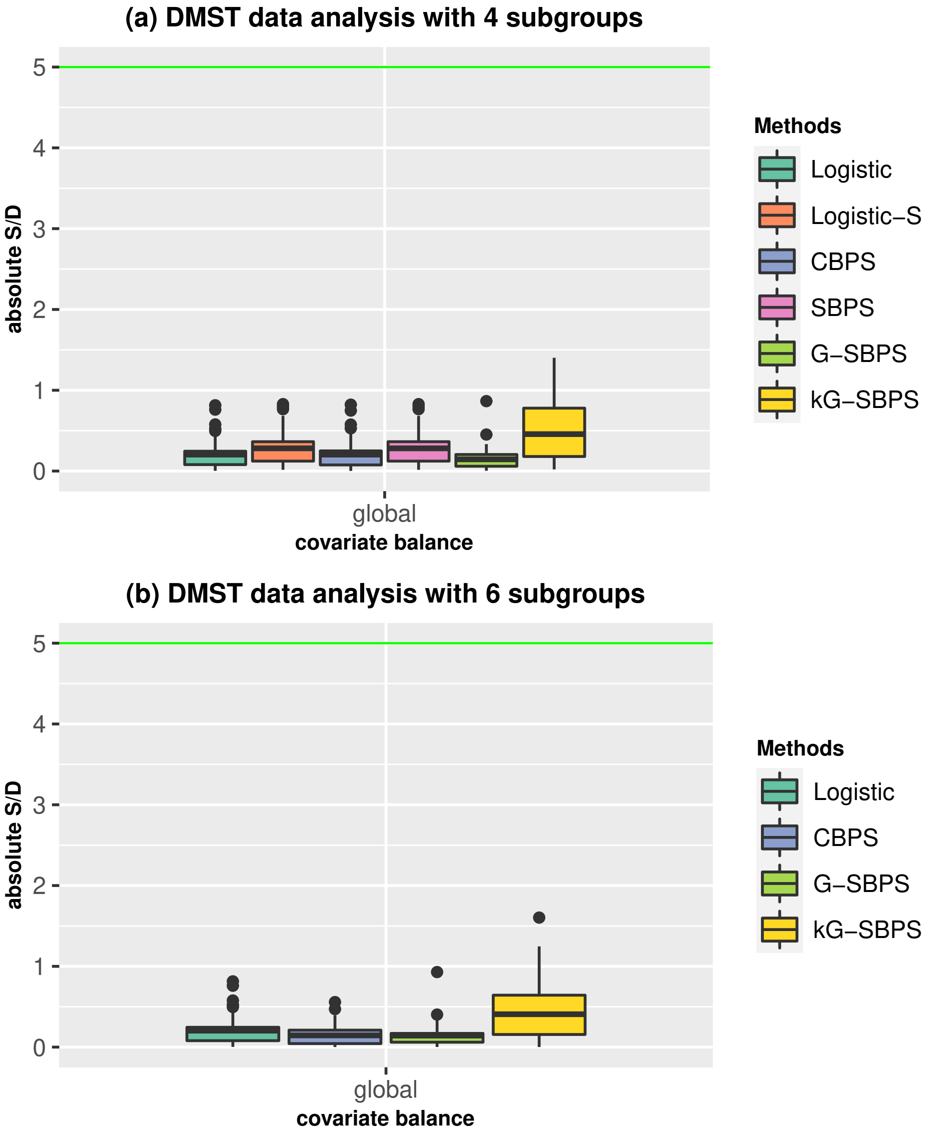

Figure S6. Boxplots of the standardized differences (S/D) of all covariates in the DSMT data analysis. Red line: 10% S/D; Green line: 5% S/D. The S/D is calculated for the overall population ("global" covariance balance).

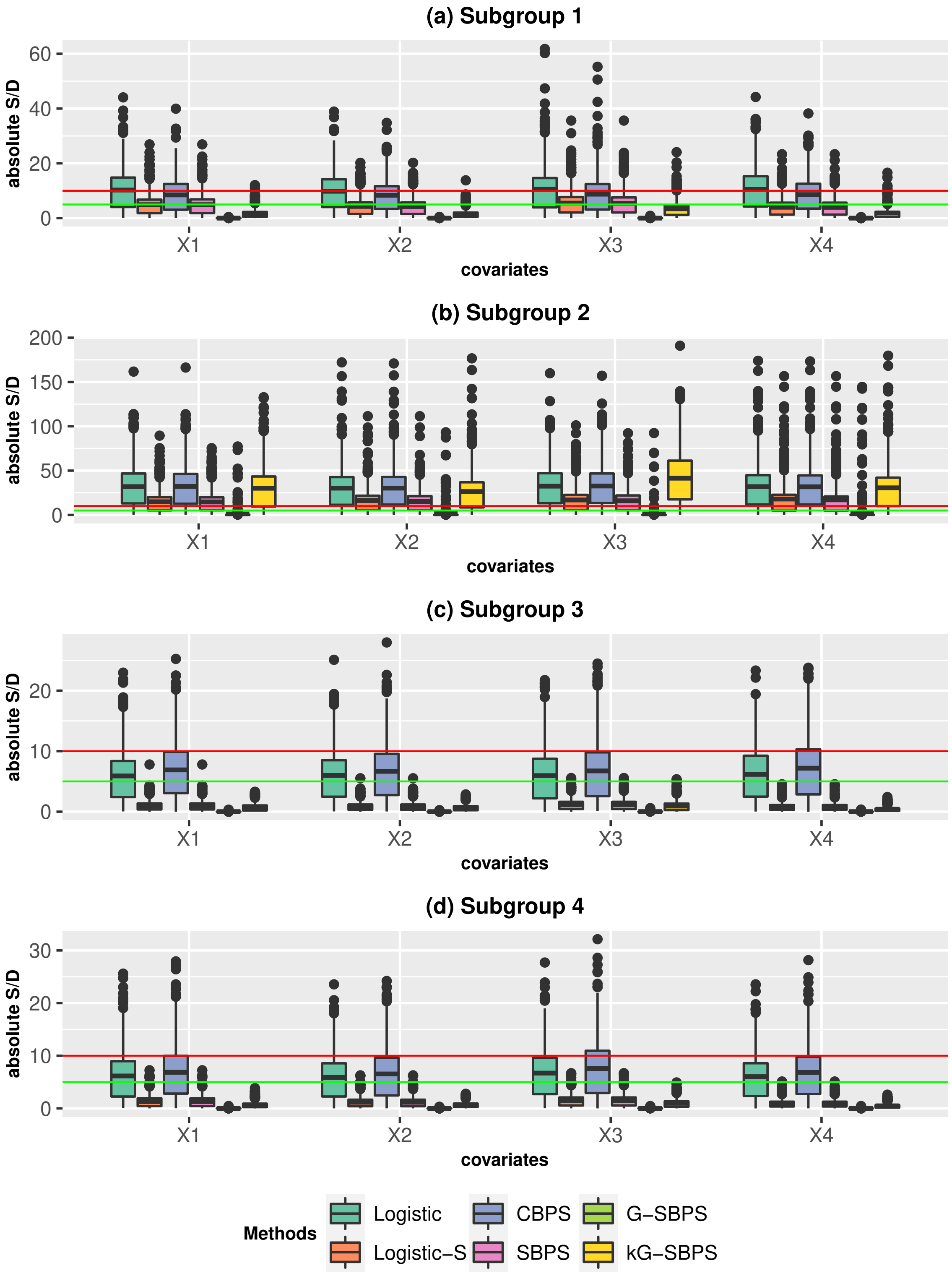

Figure S7. Boxplots of the S/D in four subgroups by different methods using IPW estimator to estimate **ATE**. The simulation scenario is Correct PS model (PS1; see Section 3.1), with 4 subgroups. $N_2 = 40$ for subgroup 2 and $N_k = 500$ for subgroup $k \in [1, 3, 4]^T$. The number of simulations is 500. Red line: 10% S/D; Green line: 5% S/D; S/D: standardized difference.

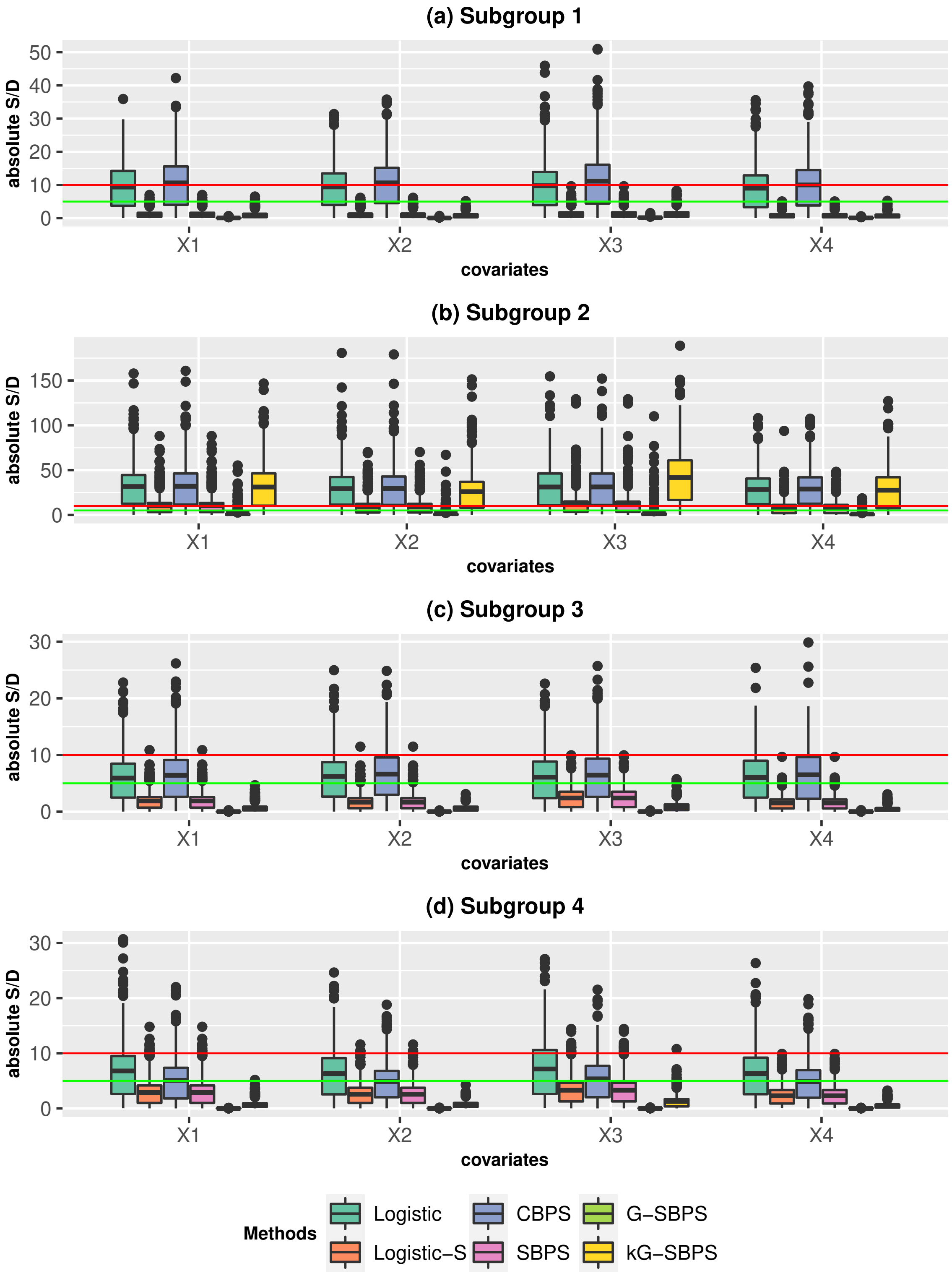

Figure S8. Boxplots of the S/D in four subgroups by different methods using IPW estimator to estimate **ATT**. The simulation scenario is Correct PS model (PS1; see Section 3.1), with 4 subgroups. $N_2 = 40$ for subgroup 2 and $N_k = 500$ for subgroup $k \in [1, 3, 4]^T$. The number of simulations is 500. Red line: 10% S/D; Green line: 5% S/D; S/D: standardized difference.

4 Simulation with 10 subgroups

4.1 Simulation Setup

We modified the simulation setup described in the main paper. Specifically, we retained the same model specification, distributional assumptions for all variables, and the procedure for defining subgroups. But here we let $K = 10$ to obtain 10 subgroups, and the sample size of each subgroup is $N_k = 250$. In addition, we adjusted the coefficient values of the propensity score models to ensure that the proportion of treated and untreated units remained approximately equal. This adjustment was made to achieve a more balanced treatment allocation. The coefficient of the two propensity score model specifications are:

PS1: (Correct PS model) $\boldsymbol{\beta} = (-0.1, -0.1, 0.1, -0.1)$.

PS2: (Misspecified PS model) $\boldsymbol{\beta} = (-0.5, -0.4, 0.2, -0.4, 0.1, 0.1)$ for ATE estimation

We used the same two outcome models (OM1, the standard outcome model and OM2, the extended outcome model) described in the main paper. The true subgroup treatment effects are still $\eta_k = -10 + 20(k - 1)/(K - 1)$, where $K = 10$.

We also evaluated the performance of the six methods in the subgroup ATE estimation, as well as overall/subgroup balance as described in the "Simulation" Section of the main paper.

4.2 Covariate balance

Overall, the results were consistent with the primary simulations with 4 subgroups. Under the correctly specified propensity score model (PS1), all methods achieved good overall balance (Fig S9, S10A, and S10B). Under the misspecified model (PS2), kG-SBPS remained the only method consistently achieving best overall and subgroup balance, while G-SBPS preserved balance for X_1 to X_4, but worse balance for interaction terms (Fig S9, S11A, and S11B). Subgroup balance were slightly higher than global balance due to smaller subgroup sizes, but G-SBPS and kG-SBPS consistently outperformed other methods. These results indicate that the conclusions from the 4 subgroup simulations generalize to settings with more subgroups, supporting the scalability of the proposed methods.

4.3 ATE estimation

Under the correctly specified propensity score model (PS1), all methods achieved small bias and RMSE across subgroups (Table S9). Logistic-S, SBPS, and G-SBPS substantially reduced bias compared with the simple Logistic and CBPS estimators, while kG-SBPS consistently achieved the lowest RMSE across both standard and extended outcome settings. The results confirm that G-SBPS and kG-SBPS provide accurate subgroup ATE estimates and maintain excellent stability even when the number of subgroups increases to 10.

When the propensity score model was misspecified (PS2), the performance differences between methods became more pronounced (Table S10). Logistic, Logistic-S, CBPS, and SBPS showed large bias, which often exceed 100%, whereas G-SBPS and especially kG-SBPS maintained near-zero bias for most subgroups. The kG-SBPS again achieved the smallest RMSE values, demonstrating strong robustness to model misspecification. Overall, these findings mirror those from the 4 subgroup simulation and indicate that both G-SBPS and kG-SBPS scale effectively to larger numbers of subgroups while preserving bias reduction and estimation stability.

Table S9: The performance of various methods to estimate **subgroup ATEs** in the simulations with the **correct PS model** with **10 subgroups**. $N_k = 250$. The number of Monte Carlo repetitions is 500.

	**Outcome**	**Subgroup**	**Logistic**	**Logistic-S**	**CBPS**	**SBPS**	**G-SBPS**	**kG-SBPS**
%Bias	Standard	**1**	-1.07	-1.34	-0.83	-1.20	-0.20	-1.84
		**2**	-0.20	-0.69	-0.19	-0.61	0.03	-0.77
		**3**	4.96	-0.53	4.97	-0.53	0.17	-0.70
		**4**	-2.63	-0.21	-2.79	-0.21	0.31	-0.15
		**5**	-1.11	1.37	-1.62	1.37	0.16	0.07
		**6**	20.63	-0.32	21.25	-0.32	0.44	-0.96
		**7**	-4.28	-0.11	-4.13	-0.11	-0.08	-0.41
		**8**	-0.83	0.26	-0.71	0.26	0.10	0.08
		**9**	0.19	-0.16	0.25	-0.20	-0.09	0.14
		**10**	-0.78	-0.14	-0.74	-0.14	-0.02	0.62
	Extended	**1**	-1.24	-0.76	-1.88	-0.82	-3.19	4.69
		**2**	-3.98	-0.07	-3.91	-0.28	-0.57	1.68
		**3**	-1.97	1.65	-1.78	1.65	0.13	-0.66
		**4**	-4.92	-1.26	-4.30	-1.26	-1.54	-2.22
		**5**	12.70	6.28	13.88	6.28	4.91	-4.52
		**6**	-1.04	1.51	-1.56	1.51	2.86	2.49
		**7**	-6.98	-2.73	-7.16	-2.73	-2.93	-0.40
		**8**	-2.01	-0.39	-2.01	-0.39	-0.54	-0.23
		**9**	0.56	-0.34	0.61	-0.38	-0.37	-0.55
		**10**	-0.78	-0.10	-0.74	-0.11	-0.13	0.33
RMSE	Standard	**1**	3.92	1.27	3.82	1.24	0.18	0.81
		**2**	3.44	0.95	3.36	0.95	0.17	0.50
		**3**	3.32	0.79	3.28	0.79	0.14	0.37
		**4**	3.01	0.50	3.02	0.50	0.13	0.32
		**5**	2.85	0.33	2.89	0.33	0.14	0.32
		**6**	2.81	0.23	2.88	0.23	0.12	0.28
		**7**	2.64	0.18	2.70	0.18	0.13	0.27
		**8**	2.66	0.27	2.73	0.27	0.13	0.27
		**9**	2.89	0.36	2.93	0.36	0.14	0.29
		**10**	3.21	0.56	3.20	0.56	0.14	0.35
	Extended	**1**	8.51	3.43	8.18	3.36	1.59	1.80
		**2**	6.53	2.40	6.36	2.37	1.40	1.11
		**3**	5.70	2.09	5.61	2.09	1.41	0.57
		**4**	4.42	1.49	4.45	1.49	1.20	0.42
		**5**	3.42	1.25	3.47	1.25	1.16	0.33
		**6**	2.64	1.16	2.67	1.16	1.13	0.26
		**7**	2.42	1.15	2.47	1.15	1.14	0.26
		**8**	2.53	1.12	2.58	1.12	1.10	0.30
		**9**	3.01	1.31	3.05	1.31	1.23	0.37
		**10**	3.94	1.53	3.92	1.53	1.25	0.51

Table S10: The performance of various methods to estimate **subgroup ATEs** in the simulations with the **misspecified PS model** with **10 subgroups**. $N_k = 250$. The true treatment effect for subgroup 1 to 10 are -10, $-70/9$, $-50/9$, $-30/9$, $-10/9$, $10/9$, $30/9$, $50/9$, $70/9$, and 10, respectively. The number of Monte Carlo repetitions is 500.

	**Outcome**	**Subgroup**	**Logistic**	**Logistic-S**	**CBPS**	**SBPS**	**G-SBPS**	**kG-SBPS**
%Bias	Standard	**1**	-116.21	4.71	-116.08	4.71	-0.004	-3.03
		**2**	-122.12	7.17	-121.55	7.17	-0.02	-3.86
		**3**	-126.44	11.24	-125.59	11.24	0.10	-3.81
		**4**	-131.60	16.19	-130.15	16.19	0.41	-5.06
		**5**	-162.63	29.32	-158.22	29.32	-0.15	-9.05
		**6**	-122.39	-7.74	-126.98	-7.74	0.40	4.89
		**7**	-125.89	2.72	-127.56	2.72	0.26	-0.18
		**8**	-129.44	0.95	-130.45	0.95	0.02	-0.78
		**9**	-117.30	-2.08	-117.98	-2.08	0.002	-1.03
		**10**	-110.35	-5.57	-110.84	-5.57	-0.02	-0.98
	Extended	**1**	154.71	1.06	154.24	1.06	5.33	5.96
		**2**	132.82	2.30	132.09	2.30	7.53	5.22
		**3**	109.84	5.13	109.03	5.13	13.33	4.04
		**4**	79.00	15.38	78.11	15.38	18.16	3.09
		**5**	102.09	60.59	100.87	60.59	60.13	-1.63
		**6**	-104.99	-77.86	-104.91	-77.86	-74.15	3.46
		**7**	-71.97	-21.56	-72.49	-21.56	-22.09	-0.47
		**8**	-94.20	-11.54	-94.81	-11.54	-12.11	-1.27
		**9**	-114.61	-11.53	-115.22	-11.53	-9.78	-2.41
		**10**	-133.53	-13.72	-134.13	-13.72	-7.75	-2.69
RMSE	Standard	**1**	11.95	2.07	11.94	2.07	0.15	0.47
		**2**	9.95	1.61	9.91	1.61	0.15	0.52
		**3**	7.65	1.55	7.61	1.55	0.14	0.40
		**4**	5.40	1.33	5.34	1.33	0.14	0.37
		**5**	3.64	0.93	3.60	0.93	0.15	0.31
		**6**	3.31	0.58	3.31	0.58	0.13	0.27
		**7**	5.04	0.43	5.09	0.43	0.13	0.24
		**8**	7.76	0.34	7.83	0.34	0.13	0.24
		**9**	9.50	0.44	9.56	0.44	0.13	0.27
		**10**	11.44	0.96	11.49	0.96	0.14	0.31
	Extended	**1**	16.72	2.23	16.69	2.23	1.55	0.96
		**2**	11.81	1.29	11.76	1.29	1.49	0.72
		**3**	7.92	1.06	7.87	1.06	1.61	0.57
		**4**	5.20	1.13	5.15	1.13	1.51	0.42
		**5**	3.74	1.32	3.72	1.32	1.46	0.33
		**6**	3.02	1.50	3.01	1.50	1.50	0.28
		**7**	3.58	1.36	3.59	1.36	1.34	0.26
		**8**	5.86	1.32	5.90	1.32	1.33	0.31
		**9**	9.33	1.50	9.38	1.50	1.37	0.42
		**10**	13.80	2.23	13.86	2.23	1.45	0.54

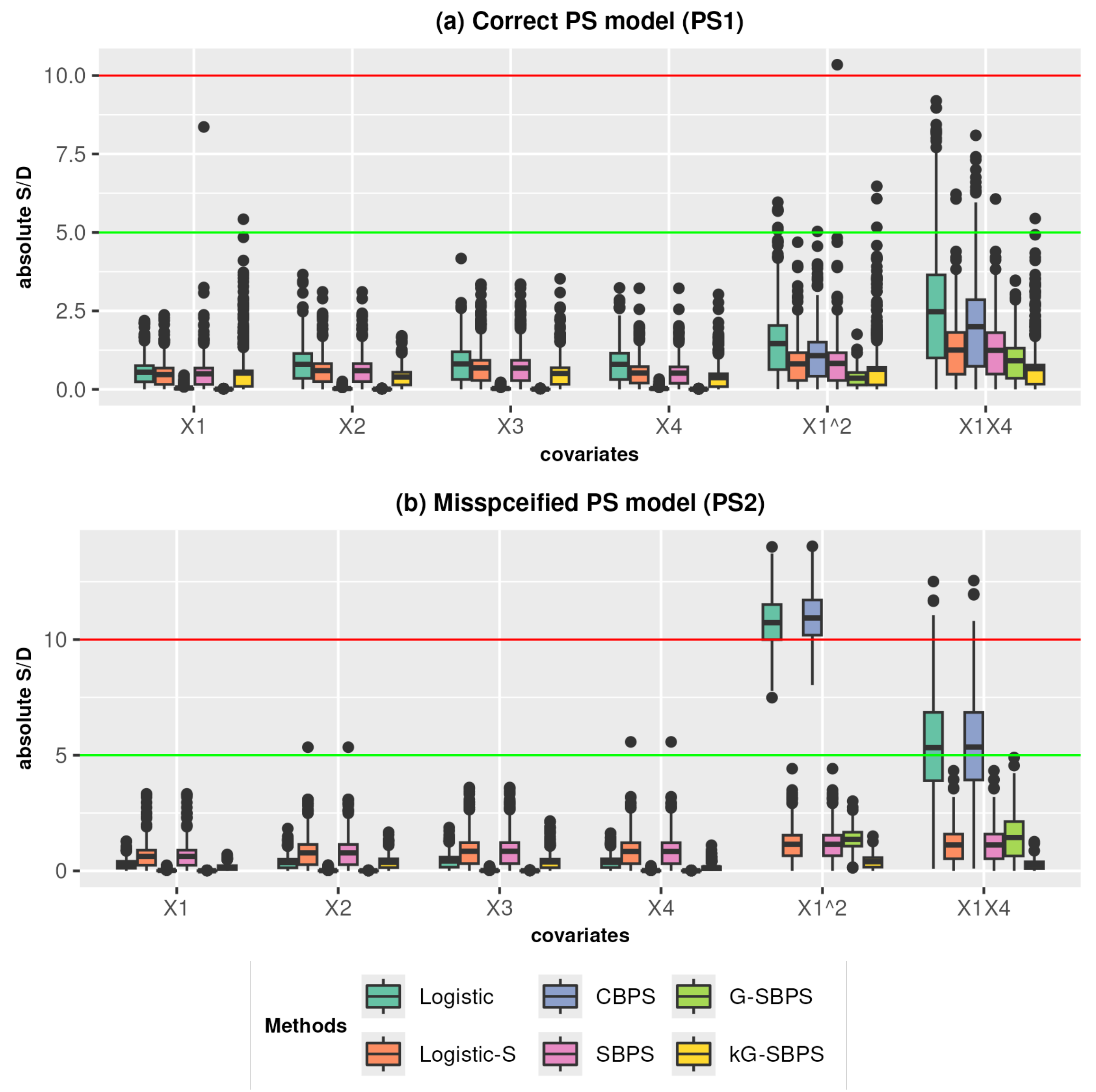

Figure S9. Boxplots of the global S/D in the estimation of **ATE** from the simulations. The data are simulated from the correct or misspecified PS model (PS1 or PS2; "Simulation with 10 subgroups"). The boxplots show the distribution of S/D from 500 Monte Carlo repetitions. The red and green horizontal lines mark the 10% and 5% S/D, respectively.

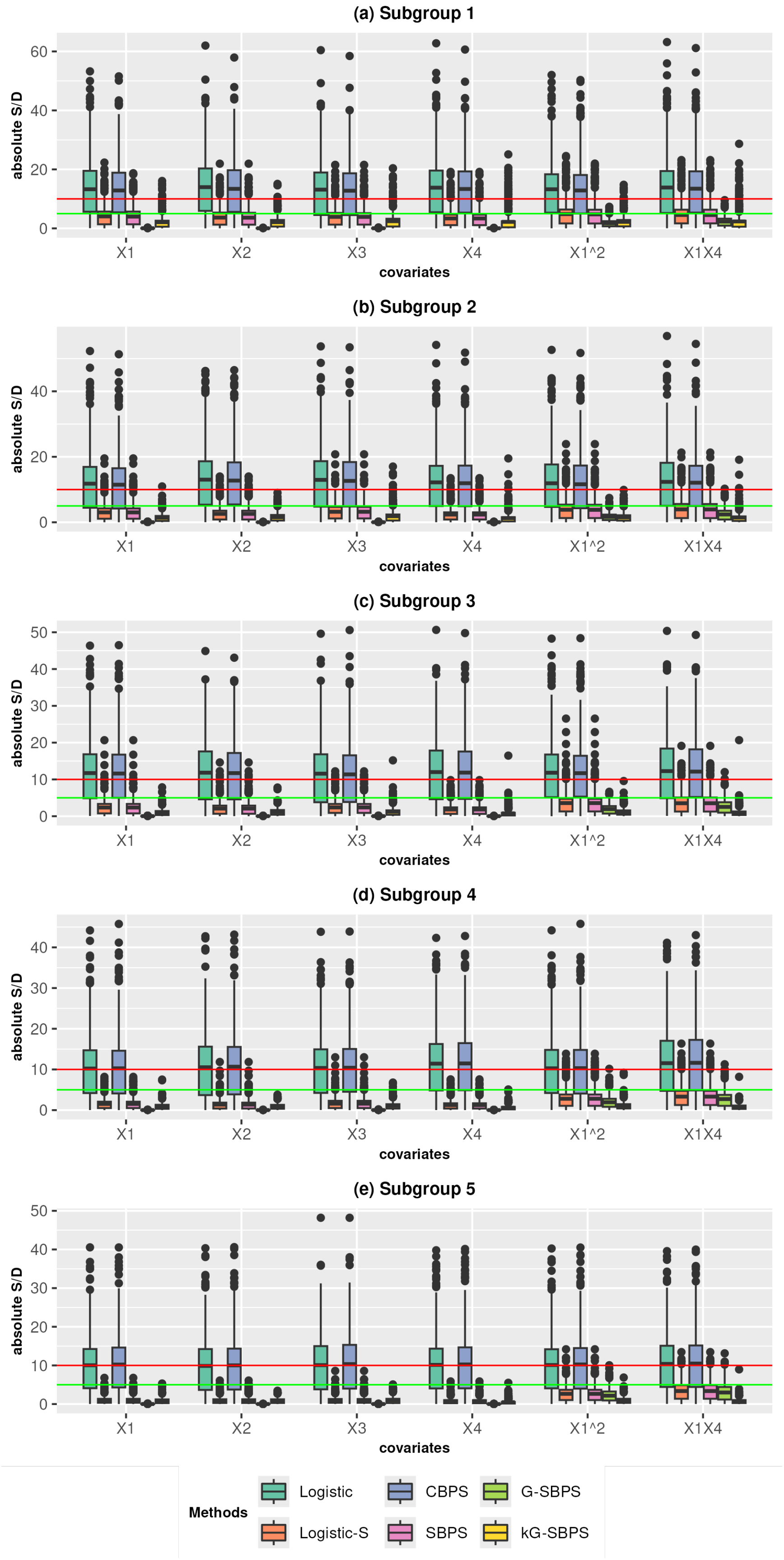

Figure S10A. Boxplots of the subgroup S/D (subgroup 1 to 5) in the estimation of **ATE** from the simulations. The data are simulated from the **correct PS model** (PS1; "Simulation with 10 subgroups"). The boxplots show the distribution of S/D from 500 Monte Carlo repetitions. The red and green horizontal lines mark the 10% and 5% S/D, respectively.

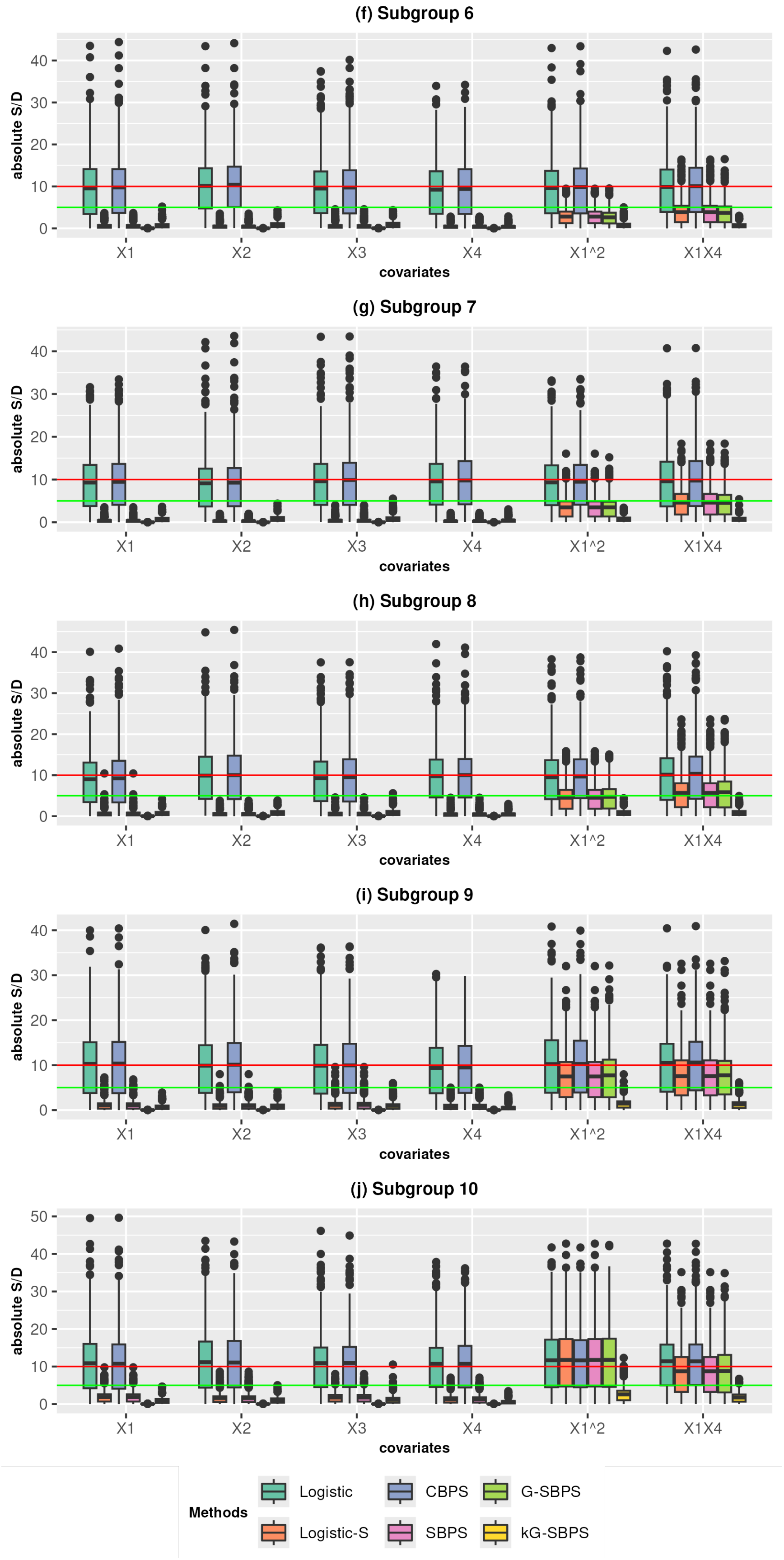

Figure S10B. Boxplots of the subgroup S/D (subgroup 6 to 10) in the estimation of **ATE** from the simulations. The data are simulated from the **correct PS model** (PS1; "Simulation with 10 subgroups"). The boxplots show the distribution of S/D from 500 Monte Carlo repetitions. The red and green horizontal lines mark the 10% and 5% S/D, respectively.

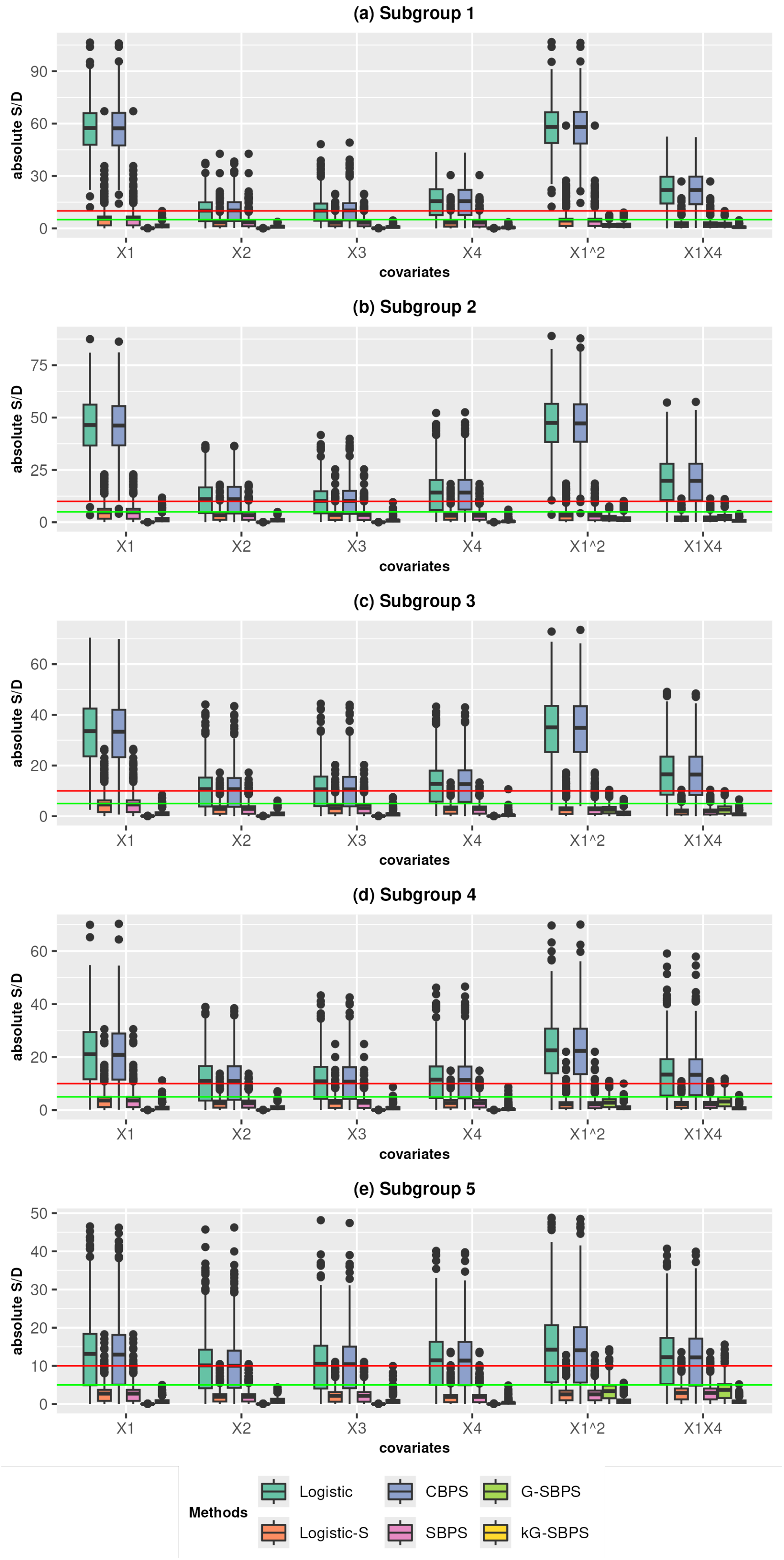

Figure S11A. Boxplots of the subgroup S/D (subgroup 1 to 5) in the estimation of **ATE** from the simulations. The data are simulated from the **misspecified PS model** (PS2; "Simulation with 10 subgroups"). The boxplots show the distribution of S/D from 500 Monte Carlo repetitions. The red and green horizontal lines mark the 10% and 5% S/D, respectively.

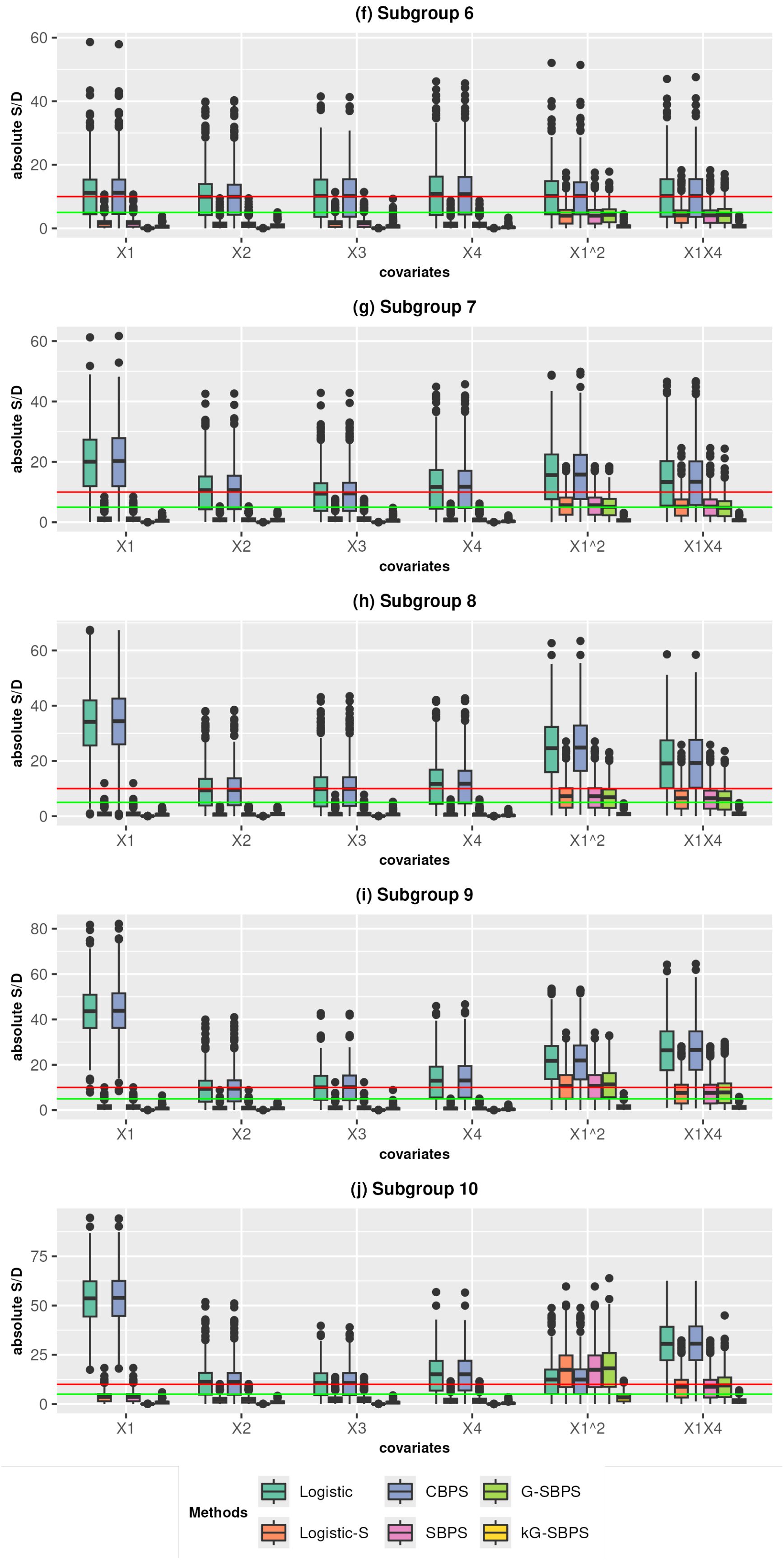

Figure S11B. Boxplots of the subgroup S/D (subgroup 6 to 10) in the estimation of **ATE** from the simulations. The data are simulated from the **misspecified PS model** (PS2; "Simulation with 10 subgroups"). The boxplots show the distribution of S/D from 500 Monte Carlo repetitions. The red and green horizontal lines mark the 10% and 5% S/D, respectively.

5 Simulation with unobserved confounders

We evaluated this scenario using only the ATE estimator. We used the misspecified propensity score model (PS2) of the primary simulation setting described in the main paper (see the "Simulation" section). However, when estimating the propensity score, the covariate X_2 was intentionally omitted to mimic the presence of an unobserved confounder.

For global balance (Fig S12), none of the methods were able to control the imbalance of X_2, as it was excluded from the propensity score model. G-SBPS and kG-SBPS achieved nearly zero imbalance for the other three covariates, while only kG-SBPS attained good balance for the quadratic and interaction terms.

In terms of subgroup balance (Fig S12), the same pattern was observed: no method adequately balanced X_2. The existing methods (Logistic, Logistic-S, CBPS, and SBPS) exhibited substantial subgroup covariate imbalance. In contrast, the proposed G-SBPS and kG-SBPS achieved good subgroup balance (standardized differences below 5%) for X_1, X_3, and X_4. Only kG-SBPS maintained good or acceptable balance for quadratic and interaction term (standardized differences below 10%).

Table S12 presents the performance of the methods under comparison for estimating subgroup ATE under the misspecified PS model, where X_2 was omitted from the PS estimation to serve as an unobserved confounder. The existing methods (Logistic, Logistic-S, CBPS, and SBPS) exhibited substantial bias and large RMSEs across subgroups, indicating poor robustness to PS model misspecification as well as unobserved confounders. In contrast, the proposed G-SBPS and kG-SBPS methods substantially reduced bias and achieved the lowest RMSEs under both outcome models. The kG-SBPS method consistently yielded nearly unbiased estimates (%Bias $<$ 15%) and the smallest RMSEs across all subgroups. These findings demonstrate the superior stability and robustness of the proposed methods, particularly kG-SBPS.

Table S11: The performance of various methods to estimate **subgroup ATEs** in the simulations with the **misspecified PS model (PS2, see Section 3.1)**. X_2 was not included in the propensity score estimation. The true treatment effect for subgroup 1 to 4 are -10, $-10/3$, $10/3$, and 10, respectively. The number of Monte Carlo repetitions is 500.

	**Outcome**	**Subgroup**	**Logistic**	**Logistic-S**	**CBPS**	**SBPS**	**G-SBPS**	**kG-SBPS**
%Bias	Standard	**1**	-200.96	114.29	-203.29	40.71	3.98	-0.67
		**2**	-262.11	206.46	-275.81	193.73	10.28	5.39
		**3**	-300.06	20.77	-285.79	20.64	-10.93	-13.01
		**4**	-196.86	-21.57	-194.17	-21.57	-4.28	-7.11
	Extended	**1**	35.76	58.34	36.62	41.79	38.12	7.08
		**2**	-0.85	224.82	-5.10	217.20	124.05	13.99
		**3**	-325.93	-84.59	-314.23	-84.68	-106.69	-18.05
		**4**	-239.64	-51.27	-236.68	-51.27	-33.70	-11.50
RMSE	Standard	**1**	20.17	16.61	20.40	12.88	0.59	0.57
		**2**	9.11	7.79	9.51	7.63	0.47	0.48
		**3**	10.22	1.03	9.76	1.03	0.47	0.58
		**4**	19.78	2.51	19.51	2.51	0.55	0.92
	Extended	**1**	4.20	8.96	4.29	5.30	4.37	0.92
		**2**	2.10	8.26	2.05	7.87	4.34	0.64
		**3**	11.07	3.01	10.68	3.02	3.69	0.73
		**4**	24.07	5.42	23.77	5.42	3.62	1.37

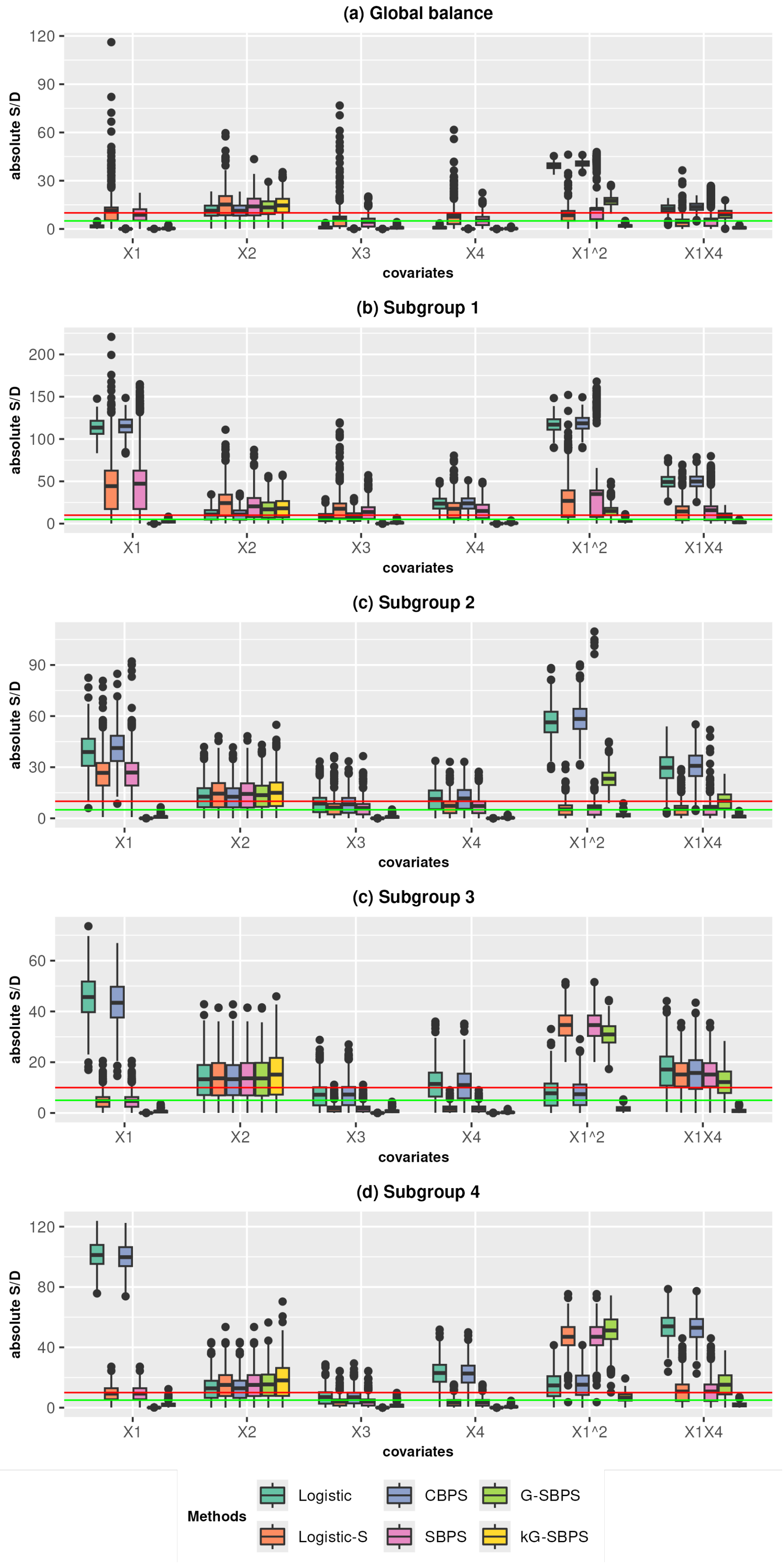

Figure S12. Boxplots of the global and subgroup S/D in the estimation of **ATE** from the simulations. The data are simulated from the **misspecified PS model** (PS2; see Section 3.1). X_2 was omitted when estimating the propensity score. The boxplots show the distribution of S/D from 500 Monte Carlo repetitions. The red and green horizontal lines mark the 10% and 5% S/D, respectively.
